# LYAR Promotes Colorectal Cancer Progression by Upregulating *FSCN1* Expression and Fatty Acid Metabolism

**DOI:** 10.1155/2021/9979707

**Published:** 2021-12-06

**Authors:** Yupeng Wu, Yu Zhou, Haiying Gao, Yajun Wang, Qingyu Cheng, Shikun Jian, Qi Ding, Wei Gu, Yanxue Yao, Jia Ma, Wenjuan Wu, Yuyun Li, Xuhui Tong, Xiaoyuan Song, Sai Ma

**Affiliations:** ^1^Department of Biochemistry and Molecular Biology, School of Laboratory Medicine, Bengbu Medical College, Bengbu, Anhui 233030, China; ^2^Department of Laboratory, The Affiliated Suzhou Hospital of Nanjing Medical University, Suzhou Municipal Hospital, Gusu School, Nanjing Medical University, Suzhou, Jiangsu 215000, China; ^3^School of Chemistry and Materials Science, University of Science and Technology of China, Hefei, Anhui 230026, China; ^4^MOE Key Laboratory for Membraneless Organelles and Cellular Dynamics, Hefei National Laboratory for Physical Sciences at the Microscale, CAS Key Laboratory of Brain Function and Disease, School of Life Sciences, Division of Life Sciences and Medicine, University of Science and Technology of China, Hefei, Anhui 230027, China; ^5^School of Pharmacy, Bengbu Medical College, Bengbu, Anhui 233030, China

## Abstract

Colorectal cancer (CRC) is a highly malignant tumor associated with poor prognosis, yet the molecular mechanisms are not fully understood. In this study, we showed that LYAR, a nucleolar protein, is expressed at a higher level in CRC tissue than in adjacent normal tissue and that LYAR expression is closely associated with distant CRC metastasis. LYAR not only significantly promotes the migration and invasion of CRC cells *in vitro*, but knockdown (KD) of LYAR in CRC cells also inhibits xenograft tumor metastasis *in vivo*. Microarray analysis of *LYAR* KD cells combined with a chromatin immunoprecipitation (ChIP) assay, gene reporter assay, and rescue experiment indicated that *FSCN1* (encoding fascin actin-bundling protein 1 (Fascin-1)) serves as a novel key regulator of LYAR-promoted migration and invasion of CRC cells. Knockdown of *FSCN1* significantly inhibits subcutaneous tumorigenesis of CRC cells and leads to the downregulation of *FASN* and *SCD*, genes encoding key enzymes in fatty acid synthesis. In summary, this study reveals a novel mechanism by which LYAR promotes tumor cell migration and invasion by upregulating *FSCN1* expression and affecting fatty acid metabolism in CRC.

## 1. Introduction

Colorectal cancer (CRC) is the third most frequently diagnosed cancer, with 1.85 million new cases in 2018, and is the second most common cause of cancer-related mortality worldwide [IARC World Cancer Report 2020] [1]. With advances such as prognostic techniques, neoadjuvant chemoradiotherapy, cytotoxic therapy, and surgical therapy, great progress in treatment has been made. The five-year survival rate for CRC, however, remains poor (~64%), and in metastatic cases, it is even worse (14%) [2, 3]. Twenty-five percent of patients with CRC already have metastases at the time of diagnosis, and 50–60% of the remainder will develop metastases later in the course of the disease [4, 5]. The formation of metastasis is a multistep process, in which tumor cells leave the primary tumor site, invade and penetrate the surrounding extracellular matrix and endothelium, enter the blood and lymph vessels, survive migration, and finally attach at a distant site, where tumor cells begin to proliferate, induce angiogenesis, evade apoptotic death, and form a new tumor [6, 7]. These distant settlements of tumor cells, metastases, are the cause of 90% of human cancer deaths [6, 8–10]. Tumor recurrence and metastasis are the main causes of death in CRC. Approximately 30–50% of patients with CRC after curative surgery will relapse [11]. However, the molecular mechanism involved in the cascade of events during invasion and metastasis of CRC is still not fully understood.

LYAR was first identified in 1993 as a novel nucleolar oncoprotein, which consists of a zinc finger motif and three nuclear localization signals [12]. However, research was stagnant until a second LYAR article was published in 2009 [13]. Subsequently, there have been very few articles related to the function of LYAR [14–18], especially in the field of tumor research. In 2014, Ju et al. demonstrated that LYAR is a transcription factor with a DNA-binding motif (GGTTAT/G) that inhibits human fetal *globin* gene expression [19]. We further reported that LYAR promotes tumor cell migration and invasion by directly binding to the LYAR-binding motif (CTAACC; reverse complement GGTTAG) in the *LGALS1* promoter to activate its expression in CRC cells [20]. *LGALS1* encodes galectin-1, which is a member of the lectin family. Galectin-1 is a homodimer of 14 kD subunits and is characterized by its affinity for glycans containing *β*-galactosides. Galectin-1 has been detected in various malignancies such as colorectal, hepatocellular, prostate, ovarian, and breast cancers. Galectin-1 activation occurs via autocrine or paracrine sugar-dependent interactions with *β*-galactoside-containing glycoconjugates and participates in various key processes of carcinogenesis such as metastasis, angiogenesis, and immunosuppression [21].

The *FSCN1* gene is located on chromosome 7p22 and encodes Fascin-1, a cytoskeleton protein with a relative molecular weight of 55kD [22]. Fascin-1 can promote the formation of filamentous pseudopodia, lamellar pseudopodia, and microspines of cell membrane after cross-linking with F-actin. This enhances the movement, metastasis, and invasion of tumor cells [23]. A mass spectrometry-based proteomic analysis predicted that *FSCN1* may play an important role in metabolism, suggesting a new role of *FSCN1* in tumors [24].

In the present study, we showed that LYAR, a key regulator of CRC, regulates a novel LYAR target, *FSCN1*, to promote the migration and invasion of CRC cells, which in turn positively regulates fatty acid metabolism. Our data demonstrated the presence of a LYAR/*FSCN1*/fatty acid metabolism axis which promoted the growth of CRC tumors *in vivo*. These findings suggest that LYAR could be used as a prognostic and therapeutic candidate target for the prevention and treatment of CRC.

## 2. Materials and Methods

### 2.1. Tissue Microarrays and Immunohistochemistry

CRC tissue arrays were purchased from Shanghai Outdo Biotech Co., Ltd., Shanghai, China. The immunohistochemistry experiment and analysis were carried out according to the procedure described in our previous literature [20]. The LYAR primary antibody (orb215217) and the anti-rabbit secondary antibody were purchased, respectively, from Biorbyt and Sigma-Aldrich. All researches involving human CRC tissues were approved by the Ethics Committee of Bengbu Medical College and were in accordance with the principles of the Declaration of Helsinki.

### 2.2. Cell Lines and Cell Culture

The HCT8, HCT15, and HCT116 human colon cancer cell lines were purchased from the Typical Culture Preservation Commission Cell Bank, Chinese Academy of Sciences. HCT8 and HCT15 was maintained on gelatinized 10 cm-plate in RPMI-1640 Medium (Gibco) supplemented with 10% FBS (fetal bovine serum) (Gibco), 100 U penicillin/100 mg streptomycin (Gibco) at 37°C, and 5% CO_2_. HCT116 cells were cultured in McCoy's 5a Medium (Gibco).

### 2.3. Silencing of *LYAR* and *FSCN1* by Transient Transfection siRNA

The siRNA sequences against human *LYAR*, *FSCN1* and nonsilencing were designed and chemically synthesized by Genepharma (Shanghai, China). HCT15 cells were transfected with 100-200 pmol siRNA using 5-10 *μ*L INTERFERin® transfection reagent (Polyplus) in a 6-well plate. After 24-48 hours, cells were collected to perform cell proliferation, cell cycle, apoptosis, colony formation, adhesion, migration, and invasion assays. The siRNAs sequences were as follows:

1. Human *LYAR*-siRNA1: 5′-GGGAGGUGAAGAAGAAUAA-3′
2. Human *LYAR*-siRNA2: 5′-GCACUCGGAAGUUGAAACA-3′
3. Human *FSCN1*-siRNA1: 5′-GCGCCUACAACAUCAAAGA-3′
4. Human *FSCN1*-siRNA2: 5′-GCCCAUGAUAGUAGCUUCA-3′

### 2.4. Microarray Analysis and RNA-Seq

Approximately 1 × 10^7^ cells were collected and lysed with TRIzol® Reagent (Life Technologies) in the RNase-free Eppendorf tube. We then submitted the samples to KangChen-Biotech Corporation (Shanghai, China) for microarray analysis using the Human 12 × 135K Gene Expression Array (Roche NimbleGen). The experiment and data analysis for microarray analysis, including RNA isolation, microarray experiment, data processing, statistical analysis, and gene ontology analysis, were done by KangChen-Biotech Corporation.

*FSCN1* siRNA and NC (negative control) were transfected into cells and cultured for 24-48 hours. Then, total RNA was isolated by the RNA pure Tissue and Cell Kit and the RNA was sequencing by the 150 bp paired method. Hisat2, samtools, and htseq-count packages were used to align 150 bp paired-end reads with hg19 (UCSC). The expression of genes and transcripts was quantified by using cufflinks tools.

We downloaded expression profiles of human colorectal cancer of TCGA and normal colon data of GTEx from UCSC Xena browser (https://xenabrowser.net/datapages/).

### 2.5. Plasmid Construction and Viral Infection

To build stable knockdown cells, the small hairpin RNAs (*LYAR*-shRNAs, *FSCN1*-shRNAs, and Control-shRNA) were designed correspondingly according to the sense and antisense sequences of the two *LYAR*-specific siRNAs and *FSCN1* siRNAs (see above) and a nonspecific siRNA and synthesized by Invitrogen (Shanghai, China). These chemically synthesized oligonucleotides were annealed to generate double-stranded oligonucleotides using the touchdown program in the PCR instrument and inserted into the *Xho* I/*Hpa* I sites in the pLentiLox 3.7 vectors, which was subsequently confirmed by sequencing.

To overexpress *FSCN1*, the human *FSCN1* coding sequence (CDS) was prepared by reverse transcription-polymerase chain reaction (RT-PCR) from normal colorectal tissues and cloned into the pLVX-IRES-mCherry Vector at *Xba* I and *BamH* I sites for overexpressing *FSCN1* in LYAR-KD cells. Lentiviruses were packaged and produced in 293T cells. The supernatant of virus production was collected and filter-sterilized to infect the corresponding cells. The stably transfected cells were sorted and collected by flow cytometry using mCherry fluorescence.

### 2.6. Cell Migration and Invasion Assays

Equal amounts of cells (1 × 10^5^ in 200 *μ*L medium without FBS) were seeded on a fibronectin-coated polycarbonate membrane insert in a Transwell apparatus (Corning Costar), and medium supplemented with 20% FBS was added to the lower chamber. After incubation for 48 h at 37°C, the insert was washed with phosphate-buffered saline, and cells on the upper surface of the insert were removed by wiping with a wet cotton swab. Cells migrating to the lower membrane surface were fixed by an equal volume mixture of methanol and acetone and stained with 0.4% crystal violet and counted under a microscope (Nikon). The invasion assay was executed as described in the migration assay using upper chamber precoated with 50 *μ*L matrigel solution that original matrigel (BD Biosciences) was diluted with FBS-free medium according to the ratio of 1 : 10.

### 2.7. Quantitative RT-PCR (RT-qPCR)

Total RNA was isolated from cells with TRIzol® Reagent (Life Technologies). cDNA was synthesized using HiScript® II 1st Strand cDNA Synthesis Kit (+gDNA wiper) (Vazyme). The RT-qPCR primers were designed by Primer Premier 6.0 software. RT-qPCR was carried out in a Rotorgene 6000 (Corbett Research) using the FastStart Universal SYBR Green Master Mix (Roche) in a final volume of 20 *μ*L. The relative quantification was executed for the following genes using *GAPDH* as an internal reference in CRC cells. Each reaction was performed in triplicate and repeated at least two times.

The primers for human *GAPDH* were as follows:

1. Forward 5′-ACCATCTTCCAGGAGCGAGA-3′
2. Reverse 5′-GTTCACACCCATGACGAACATG-3′

The primers for human *LYAR* were as follows:

1. Forward 5′-GGAGGCACTCGGAAGTTGAAA-3′
2. Reverse 5′-GTTCCTCTTCGGATCTGTGATG-3′

The primers for human *FSCN1* were as follows:

1. Forward 5′-CTGCTACTTTGACATCGAGTGG-3′
2. Reverse 5′-GGGCGGTTGATGAGCTTCA-3′

The primers for human *FASN* were as follows:

1. Forward 5′-GCTGGAAGGAGGAAGAGGTT-3′
2. Reverse 5′-CTCGAGTGGTCCGTGAGTTT-3′

The primers for human *SCD* were as follows:

1. Forward 5′-CTCAGTTCCTACGCTTCGCAT-3′
2. Reverse 5′-GTCGAGGTCAGTGAACAGCA-3′

### 2.8. Western Blotting

Cell extracts were prepared using the lysis buffer (Beyotime) from the CRC cell lines. Cell lysates (20-50 *μ*g) were loaded and separated by 10-15% sodium dodecyl sulphate-polyacrylamide gel (Bio-Rad) and transferred onto nitrocellulose membranes (Millipore). After 1 h blocking with 5% nonfat milk (or 3% BSA (bovine serum albumin)) blocking solution prepared with PBST (1 × PBS with 0.1% Tween-20), the membranes were incubated overnight at 4°C with the primary antibodies against LYAR (orb215217, Biorbyt), FSCN1 (ab220195, Abcam), FASN (10624-2-AP, Proteintech), SCD (2794S, CST), and GAPDH (M171-3, MBL) as the internal control and followed by incubation with the secondary antibody (Sigma-Aldrich) for 1 h at room temperature. The specific bands were visualized with Thermo SuperSignal® West Pico Chemiluminescent Substrate (Thermo Fisher Scientific Inc.).

### 2.9. Luciferase Reporter Assay

The *FSCN1* promoter region with a 496-bp sequence containing the specific DNA-binding motif for LYAR was amplified from the genomic DNA by PCR. Subsequently, the 496-bp DNA fragment product was subcloned into pGL3-Basic (Promega) to construct a luciferase reporter plasmid (pGL3-*FSCN1*-496bp-LYAR-wildetype), and the sequence was confirmed by double-direction sequencing (Invitrogen). Based on the pGL3-*FSCN1*-496bp-LYAR-wildetype plasmid, the pGL3-*FSCN1*-496bp-LYAR-mutant plasmid was constructed using the Muta-direct™ site-directed mutagenesis kit (SBS Genetech) for mutating the LYAR binding site. Luciferase reporter assays were performed as described previously [25].

### 2.10. Chromatin Immunoprecipitation (ChIP)

ChIP assays were performed following standard ChIP procedures [26]. Chromatin fractions from HCT15 cells were immunoprecipitated with LYAR primary antibody (a kind gift from the State Key Laboratory of Pharmaceutical Biotechnology, School of Life Sciences, Nanjing University, Nanjing, China). Normal rabbit immunoglobulin G (IgG, Beyotime) served as the controls. The ChIP samples were analyzed by quantitative real-time PCR using the FastStart Universal SYBR Green Master Mix (Roche) and specific primers (Table S2) spanning the *FSCN1* promoter. A standard curve was prepared for each set of primers using serial titrations of the input DNA. The percentage of ChIP DNA was calculated relative to the input DNA from primer-specific standard curves using the Rotor-Gene 6000 Series Software 1.7. Each experiment was performed at least two independent times.

### 2.11. Rescue Experiments

The supernatant of virus production of pLVX-IRES-mCherry (empty vector) and pLVX-IRES-mCherry-*FSCN1*-OE (exogenous expression of *FSCN1*) were used, respectively, to infect the stable *LYAR*-KD and *LYAR*-Control (only infected by empty vector) cells. Those stably infected cells were sorted and collected using mCherry fluorescence by flow cytometry, followed by cell migration and invasion assays (see above).

### 2.12. Animal Experiments

Mice were purchased from and housed at Suzhou University. All animal studies were approved by the ethical regulations of Institutional Animal Care and Use Committee (IACUC) of Suzhou University. For the tumor metastasis assay, stable HCT15 clones (Control or *LYAR*-KD1) were injected into six-week-old female NOD/SCID mice. For each group, 10 mice were injected with 1 × 10^6^ cells per animal via the tail vein. The mice were sacrificed 6 weeks after injection, and the lung and liver metastases were examined. Metastastic nodules in lung and liver tissues were fixed in Bouin's solution (Applygen), and the number of metastases was counted. The tumor samples were embedded in paraffin, cut into 5 *μ*m sections, and stained with hematoxylin and eosin (H&E).

For the tumor xenograft assay, 8 six-week-old female nude mice were processed by subcutaneous implantation of 1 × 10^6^ HCT15 cells expressing either control shRNA or *FSCN1*-shRNA. Mice were maintained for 30 days, and tumor volumes were measured at indicated time points. At the end of experiments, mice were euthanized and xenograft tumors were dissected for further analyses.

### 2.13. Statistical Analysis

All statistical analyses were performed using SPSS 22.0 software (SPSS Inc. Chicago, IL, USA). The experimental results were statistically evaluated using *Student'st-test* for comparisons between two groups or ANOVA for comparisons between more than two groups. Kaplan-Meier survival curves were generated to determine the relationship between *LYAR* levels and the overall survival of CRC patients, and the differences between the curves were calculated using the log-rank test. Multiple Cox proportional hazards regression was carried out to identify the independent factors with a significant impact on patient survival. *p* values < 0.05 were considered statistically significant.

## 3. Results

### 3.1. LYAR Is Highly Expressed in Human CRC Tissue and Promotes CRC Metastasis

**Figure 1:**
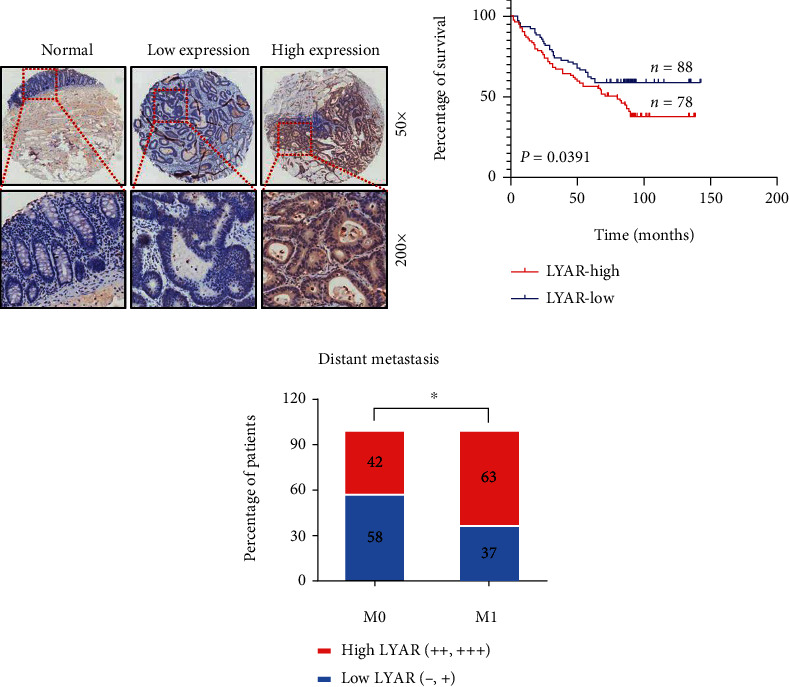
LYAR is highly expressed in human CRC tissues. (a) Immunohistochemical staining showing the LYAR protein (brown) in adjacent normal colorectal tissue and carcinoma tissue from CRC patients. Representative micrographs are shown at the original magnification (50x and 200x). Low and high LYAR expressions were defined based on immunostaining scores [20]. (b) The prognostic curve of LYAR. (c) The percentage of patients with different metastasis statuses (M0: no regional or distant metastasis; M1: regional or distant metastasis). ^∗^*p* < 0.05.

Based on data from the Oncomine database (https://www.oncomine.org/) and a previous study [20], we found that LYAR was highly expressed and promoted cell mobility in CRC cells. However, the relationship between the high expression level of LYAR and the survival rate of CRC patients is unclear. To answer this question, we performed immunohistochemistry (IHC) analysis of LYAR on tissue arrays of 166 paraffin-embedded adjacent sections of normal colorectal tissues and CRC tissues. Tissues that displayed moderate or strong immunostaining were classified as having high LYAR expression and those that displayed negative or weak immunostaining as having low LYAR expression. There was strong LYAR staining in only 4.8% (8/166) of the adjacent normal epithelial tissues, whereas ~47.0% (78/166) of total CRC tissues had high expression levels of LYAR (Figure 1(a), Table 1, and Table S1). Furthermore, high LYAR expression was significantly correlated with poor prognosis (Figure 1(b)), which was consistent with the survival curve analysis of CRC data from The Cancer Genome Atlas (TCGA) database (Figure S1). Notably, LYAR expression was positively correlated with a higher metastasis status (Figure 1(c) and Table S1). Taken together, these results indicate that LYAR is highly expressed in CRC tissues, particularly metastatic tissues. This suggests that LYAR may be involved in metastasis of CRC cells, with the potential to be a novel prognostic biomarker for CRC.

**Figure 2:**
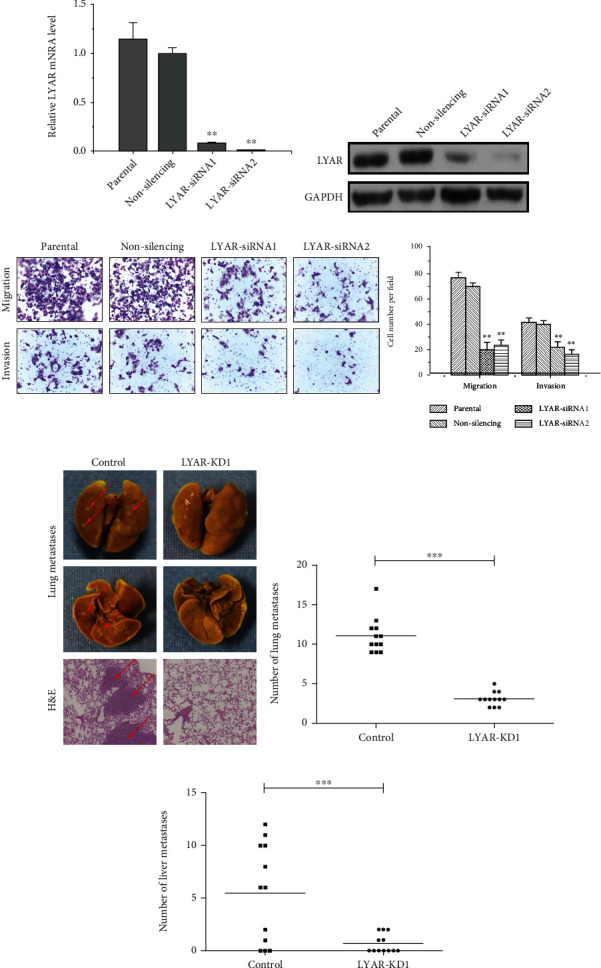
LYAR promotes metastasis of CRC cells. (a) Quantitative RT-PCR analysis of *LYAR* expression normalized to *GAPDH* expression in LYAR knockdown HCT15 cells. Results shown are the mean ± standard deviation (*n* = 3). ^∗∗^*p* < 0.01 compared with the nonspecific siRNA control. (b) Western blot assay showing LYAR protein expression in *LYAR* knockdown HCT15 cells. GAPDH served as the loading control. (c) At left, representative photos of haptotactic migration assay and matrigel chemoinvasion assay using *LYAR* knockdown HCT15 cells. Original magnification, 200x. At right, the percentage of migrated and invaded *LYAR* knockdown HCT15 cells compared to the control. ^∗∗^*p* < 0.01. (d) Representative images of metastastic nodules (indicated with arrows) in lung tissues after Bouin's fixation (upper four panels) and hematoxylin and eosin (H&E) staining of tissue sections (lower two panels). (e) Visible lung metastasis counts. ^∗∗∗^*p* < 0.001. (f) Visible liver metastasis counts. ^∗∗^*p* < 0.01.

LYAR has zinc finger DNA-binding motifs and binds to DNA at the GGTTAT/G consensus sequence [12, 19]. To perform a systematic analysis and to take our previous research a step forward, we verified that LYAR promoted migration and invasion of HCT15 cells *in vitro* (Figures 2(a)–2(c) and Figure S2-4). More importantly, we found that LYAR enhanced the lung and liver metastasis of CRC cells through a mouse tail vein assay *in vivo*. (Figures 2(d)–2(f)).

### 3.2. FSCN1 Is a Novel Target of LYAR

**Figure 3:**
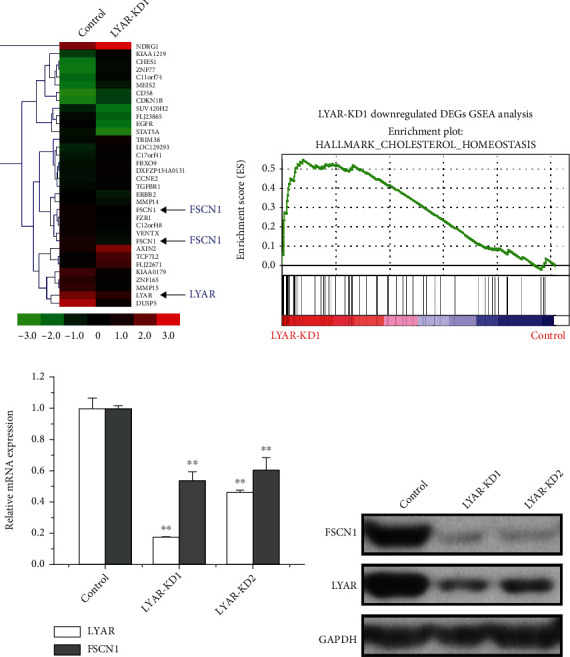
*FSCN1* is a novel target of LYAR. (a) Heatmap representation of gene expression for *LYAR* and *FSCN1* (indicated with arrows) and selected other genes in the *LYAR* knockdown or control HCT15 cells. Color represents expression values, with green indicating low, black indicating medium, and red indicating high expression. (b) Gene Set Enrichment Analysis (GSEA) plots of RNA-Seq in *LYAR* knockdown HCT15 cells. (c) Quantitative real-time PCR analysis of *LYAR* and *FSCN1* from *LYAR* knockdown or control HCT15 cells. Results shown are the mean ± standard deviation (*n* = 3). ^∗∗^*p* < 0.01. (d) Western blot assay showing LYAR and FSCN1 protein expression in *LYAR* knockdown HCT15 cells. GAPDH served as the loading control. KD1 and KD2 refer to stable *LYAR* knockdown lines *LYAR*-siRNA1 and *LYAR*-siRNA2, respectively.

Heterogeneity is a distinctive feature of solid tumors. Therefore, we speculated that there is more than one molecular mechanism by which LYAR promotes CRC migration and invasion, in addition to upregulating *LGALS1*. In order to comprehensively identify regulatory roles of LYAR in metastatic CRC, a whole-genome microarray analysis of gene expression was performed in *LYAR* knockdown (KD) and control HCT15 cells (Figure 3(a)). Gene Set Enrichment Analysis (GSEA) showed that differentially expressed genes (DEGs) in the *LYAR*-KD line were significantly enriched in cholesterol homeostasis, a lipid metabolism-related pathway (Figure 3(b)). We then screened for genes associated with metastasis and metabolism among the DEGs. The upstream promoter region (2000 bp upstream of the transcription start site) of each candidate gene was searched for the GGTTAT/G motif, which is the consensus DNA-binding site of LYAR. Candidate genes with this motif were selected for further analysis in HCT15 cells. Quantitative RT-PCR (RT-qPCR) revealed that the expression of *FSCN1*, one of the candidate genes, was consistently lower in *LYAR*-KD cells compared to the control (Figure 3(c)). Downstream involvement of *FSCN1* in *LYAR*-regulated CRC would be a novel finding; we therefore conducted further experiments to verify this relationship between *LYAR* and *FSCN1*. We detected *FSCN1* expression after *LYAR* knockdown by siRNA in all three cell lines and found that *FSCN1* was only downregulated in HCT15 (Figure 3(d) and Figure S5). We therefore performed the subsequent experiments only in HCT15 cells. Together, these results indicated that *FSCN1* is a potential target of LYAR in CRC cells.

### 3.3. LYAR Binds to the *FSCN1* Promoter in CRC Cells

**Figure 4:**
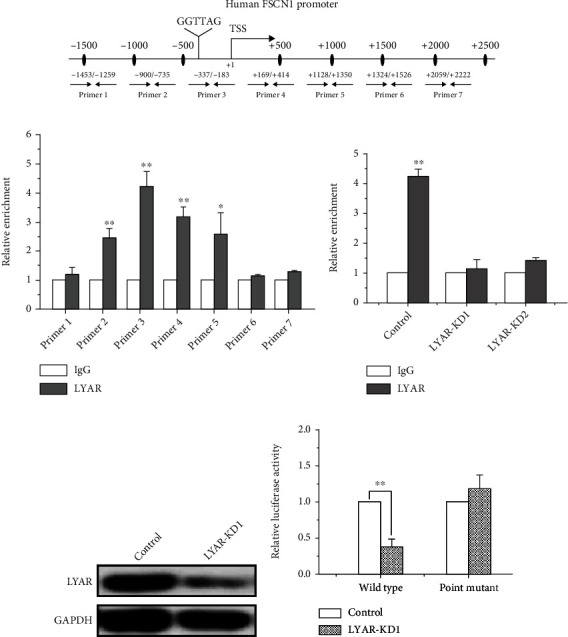
LYAR binds to the *FSCN1* promoter and directly activates the expression of *FSCN1*. (a) A schematic diagram showing seven primer pairs spanning the *FSCN1* promoter that were designed for ChIP. (b) ChIP analysis of LYAR on the *FSCN1* promoter in HCT15 cells. Normal rabbit IgG served as the control. Results are shown as the mean ± standard deviation (*n* = 3). ^∗∗^*p* < 0.01 and ^∗^*p* < 0.05 compared to the IgG control. (c) ChIP analysis of LYAR on the *FSCN1* promoter in *LYAR* knockdown and control HCT15 cells. Normal rabbit IgG served as the control. Results are shown as the mean ± standard deviation (*n* = 3). ^∗∗^*p* < 0.01 compared to the IgG control. (d) Western blot assay showing LYAR protein expression in *LYAR* knockdown HCT15 cells. GAPDH served as the loading control. (e) Luciferase reporter analyses of wild-type *FSCN1* promoter (“Wild type”) and mutant *FSCN1* promoter (“Point mutant”) in *LYAR* knockdown HCT15 cells. Results are shown as the mean ± standard deviation (*n* = 3). ^∗∗^*p* < 0.01 compared with the scrambled shRNA control.

We identified a consensus DNA-binding motif of LYAR (GGTTAG) at -237 bp upstream of the *FSCN1* gene transcriptional start site (Figure 4(a) and Table S2). Chromatin immunoprecipitation (ChIP) analysis spanning the promoter of the *FSCN1* gene was performed to determine if LYAR binds to the *FSCN1* promoter and regulates its expression. LYAR indeed bound to the *FSCN1* promoter region between -337 and -183 bp in HCT15 cells (Figure 4(b)). When *LYAR* was knocked down in HCT15 cells with shRNAs, LYAR enrichment on the *FSCN1* promoter was significantly reduced (Figure 4(c)). Furthermore, a luciferase reporter assay showed that the extent of LYAR binding to the *FSCN1*promoter corresponded to changes in LYAR expression in HCT15 cells transfected with a wild-type *FSCN1* promoter-driven luciferase construct (Figures 4(d) and 4(e)). Mutating the LYAR binding motif from GGTTAG to GACTAG abolished corresponding *FSCN1* promoter activity changes (Figures 4(d) and 4(e)). In summary, these results demonstrated that LYAR directly regulates *FSCN1* gene expression and that the LYAR-binding motif (GGTTAG) in the promoter of *FSCN1* plays a critical role in LYAR-mediated transcriptional activation of *FSCN1*.

### 3.4. Ectopic Expression of FSCN1 Restores Migration and Invasion Potential of *LYAR*-KD Cells

**Figure 5:**
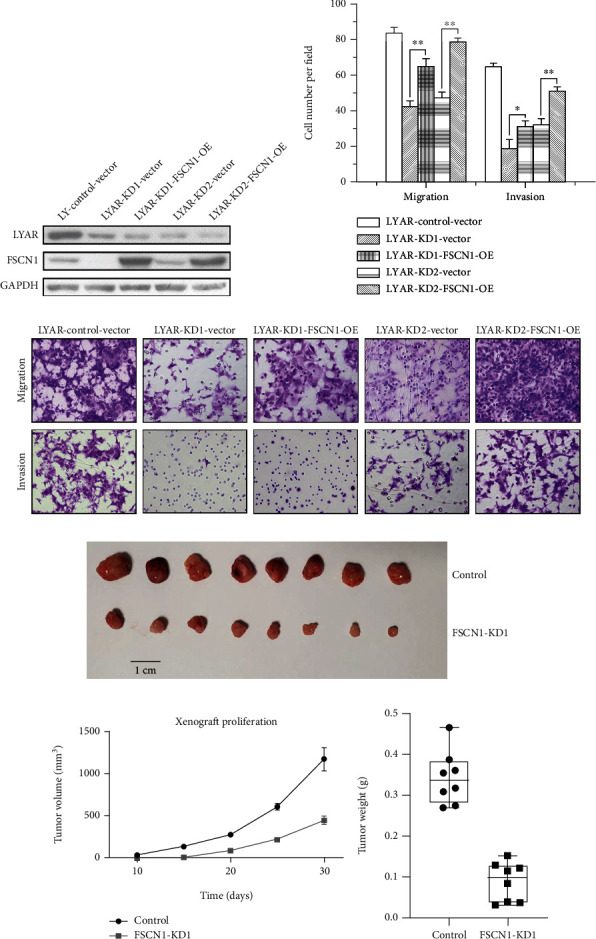
*FSCN1* overexpression restores the metastatic potential of *LYAR* knockdown cells. (a) Western blot assay showing LYAR and FSCN1 protein expression in *LYAR* knockdown (KD) HCT15 cells with and without *FSCN1* overexpression (OE). GAPDH served as the loading control. (b) Representative photos of haptotactic migration assay and matrigel chemoinvasion assay using *LYAR* knockdown HCT15 cells with and without *FSCN1* overexpression. Original magnification, 200x. (c) Results of the migration and invasion assays shown in (b). Data are shown as the mean ± standard deviation (*n* = 3). ^∗∗^*p* < 0.01 and ^∗^*p* < 0.05 compared with the empty vector control. (d) Xenografted tumors of negative control (NC) cells and stable *FSCN1*-KD HCT15 cells (n = 8). (e) Tumor volumes over time. (f) Xenografted tumors were resected and weighed at the end of the experiment. Results are shown as the mean ± standard deviation (*n* = 8). ^∗^*p* < 0.05, ^∗∗^*p* < 0.01, and ^∗∗∗^*p* < 0.001. OE: stable *FSCN1* overexpression in *LYAR* knockdown (KD) HCT15 cells.

*FSCN1* has been reported to increase CRC migration and invasion in cell cultures and cause cell dissemination and metastasis *in vivo* [27]. In this study, we found that knockdown of *LYAR* by shRNAs in HCT15 cells led to downregulation of *FSCN1* (Figures 3(c) and 3(d)) and that the cell migration and invasion potential were correspondingly decreased (Figures 2(c) and S4). To investigate the role of *FSCN1* in LYAR-promoted CRC cell migration and invasion, rescue experiments were performed in *LYAR*-KD cells in which *FSCN1* was overexpressed by a stably transfected *FSCN1*-coding sequence. Overexpression of *FSCN1* and knockdown of *LYAR* were confirmed by Western blot in HCT15 cells (Figure 5(a)). Compared with the control *LYAR*-KD cells (*LYAR*-KD-Vector), the migration and invasion potential of the HCT15 cells with *FSCN1* overexpression was significantly increased (Figures 5(b) and 5(c)), indicating that exogenous expression of *FSCN1* partially restored cell migration and invasion potential of *LYAR*-KD cells. To test this effect *in vivo*, stable *FSCN-*KD or control HCT15 cells were subcutaneously adoptively transferred to nude mice. Xenografts in the *FSCN1*-KD group were significantly inhibited compared to those in the control group (Figures 5(d)–5(f)). In summary, these results indicated that *FSCN1* plays a key role in mediating LYAR-promoted CRC cell migration and invasion.

### 3.5. Reduction of FSCN1 Expression in CRC Cells Inhibits the Expression of Some Key Enzymes in Fatty Acid Metabolism

**Figure 6:**
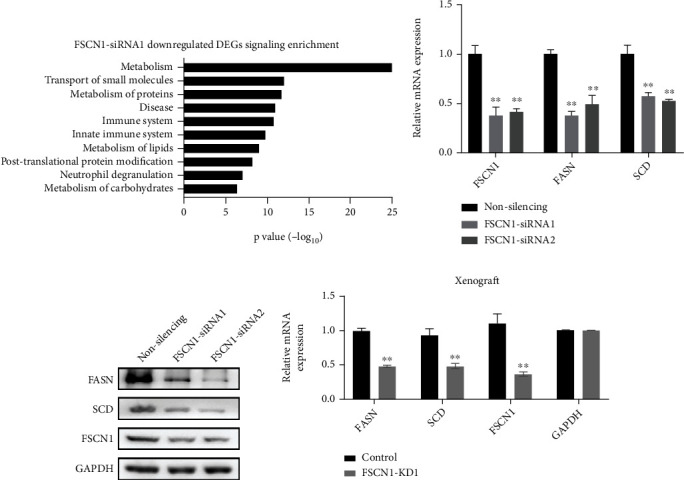
Reduction of *FSCN1* expression in CRC cells inhibits the expression of some key enzymes in fatty acid metabolism. (a) Downregulated genes (fold change > 2) were enriched in several pathways (based on the Reactome pathway database). (b) Quantitative real-time PCR analysis of *FSCN1*, *FASN*, and *SCD* from *FSCN1* knockdown or control HCT15 cells. Results are shown as the mean ± standard deviation (*n* = 3). ^∗∗^*p* < 0.01 compared with the control. (c) Western blot assay showing FSCN1, FASN, and SCD protein expression in *FSCN1* knockdown HCT15 cells. GAPDH served as the loading control. (d) Quantitative real-time PCR analysis of *FASN*, *SCD*, and *FSCN1* in *FSCN1* knockdown or control HCT15 cells from xenograft. ^∗∗^*p* < 0.01 compared with the control.

To clarify the mechanism of *FSCN1* in promoting tumor invasion and metastasis, RNA-Seq was used to search for potential *FSCN1* targets and signaling pathways. In the *FSCN1*-KD line, metabolic pathways, including lipid metabolism, were enriched (Figure 6(a)). Because our previous analysis showed that *LYAR* knockdown caused a reduction in cholesterol homeostasis, which is also a lipid metabolism pathway (Figure 3(b)), we further investigated the influence of *FSCN1* inhibition on key enzymes in lipid metabolism. Among the lipid metabolism-related genes, the expression of fatty acid synthase (*FASN*) and stearoyl-CoA desaturase (*SCD*) were consistently decreased in *FSCN1*-KD HCT15 cells compared to the control at both the mRNA (Figure 6(b)) and protein (Figure 6(c)) levels. It was previously reported that *de novo* synthesis of palmitic acid from acetyl-CoA is mainly catalyzed by *FASN*; moreover, saturated fatty acids (SFAs) are converted into monounsaturated fatty acids (MUFAs) by *SCD*, and their chains are elongated by elongases [28]. These data suggest that *FSCN1* positively regulates fatty acid metabolism.

In xenograft samples, *FASN* and *SCD* were also downregulated in the *FSCN1*-KD line (Figure 6(d)). Intriguingly, the analysis of TCGA CRC data revealed that the expression levels of *LYAR*, *FSCN1*, *FASN*, and *SCD* in CRC tissues were all higher than those in normal tissues (Figure S6). This suggests that LYAR may promote CRC progression by upregulating *FSCN1* expression and subsequent fatty acid metabolism, which is logically consistent with the results of our *in vitro* experiments (Figures 6(b)–6(d)). In conclusion, our data demonstrated the presence of a LYAR/*FSCN1*/fatty acid metabolism axis which promoted the growth of CRC tumors *in vivo*.

## 4. Discussion

The formation of metastasis is a complex, multistep process, and the molecular mechanisms of CRC metastasis are still not fully understood. In recent years, some studies have found that LYAR plays an important role in different biological processes, such as RNA synthesis and the development of erythroid cells [14–18, 29, 30]. LYAR may be involved in the *MYCN* signaling pathway in the development of medulloblastoma [31] and induces neuroblastoma cell proliferation and survival [32], and LYAR together with other five genes (*PDIA3*, *NOP14*, *NCALD*, *MTSS1*, and *CYP1B1*) can be used as potential prognostic biomarkers for curative and postoperative supportive therapies for ovarian cancer [33]. However, aside from these, there have been no other reports on the function of LYAR in cancer.

We previously found that LYAR was expressed at a higher level in metastatic CRC tissues and that it promotes tumor cell migration and invasion by upregulating *LGALS1* expression in CRC cell lines [20]. However, that study lacked *in vivo* experiments; the potential of LYAR to promote metastasis could not be verified, and retrospective analysis could not be conducted on the survival of clinical CRC patients to hypothesize whether LYAR was an independent prognostic factor. In addition, the heterogeneity of solid tumors makes it unlikely that upregulating the expression of *LGALS1* is the only molecular mechanism by which LYAR promotes CRC migration and invasion among all colorectal cancers with high LYAR expression.

In this study, we used large-scale immunohistochemical tissue microarrays and found that LYAR is highly expressed in CRC tissues. This high expression is positively correlated with the metastatic rate of CRC and is significantly correlated with poor CRC prognosis. Moreover, we performed a genome-wide expression profile analysis to further study heterogeneity in molecular mechanisms through which LYAR participates in the progression of colorectal cancer, demonstrating that LYAR plays a role in the promotion of CRC invasion and metastasis by upregulating *FSCN1* expression. However, we detected the expression of *FSCN1* after *LYAR* siRNA knockdown in three different cell lines and found that *FSCN1* was only downregulated in HCT15. This suggested that LYAR regulates *FSCN1* expression in only a subset of colorectal cancers. This phenomenon may be due to differences in race, gender, and age of the patients from whose tissue different colorectal cancer cell lines are derived; these can lead to differences in molecular features between cell lines. This result again illustrates that solid tumors exhibit significant heterogeneity.

*FSCN1* has recently been shown to promote cancer cell migration and invasion through its role in formation of cellular protrusions such as filopodia and invadopodia [34]. Forced expression of *FSCN1* in CRC cells increased their migration and invasion *in vitro* and caused cell dissemination and metastasis *in vivo* [27]. Another recent study showed that imipramine, a novel *FSCN1* inhibitor, has been confirmed to have significant antitumor effects both *in vivo* and *in vitro*, which lays the foundation for molecular targeted therapy of serrated adenocarcinoma (SAC) and other *FSCN1*-overexpressing tumors [35]. In this study, we revealed that LYAR positively regulates *FSCN1* expression. We also found that *LYAR* knockdown in CRC cells led to the reduction of *FSCN1* levels and inhibited metastasis to the lungs and liver in NOD/SCID mice. This is consistent with the report that forced expression of *FSCN1* in CRC cells caused cell dissemination and metastasis *in vivo*.

There is previous evidence in the literature of *FSCN1* involvement in tumor metabolism. In lung cancer, *FSCN1* promotes cancer growth and metastasis by enhancing glycolysis and *PFKFB3* expression through *YAP1* activation [36]. In the context of *PIK3CA* mutation or amplification, high expression of *FSCN1* is associated with poor prognosis and radiotherapy response in cancer patients; mutant *PIK3CA* (E542K and E545K) can enhance glucose metabolism and cell proliferation in cancer cells [37, 38]. In the present study, separate from glucose metabolism, we demonstrated that *FSCN1* was related to lipid metabolism in CRC. *FSCN1* knockdown reduced the expression of *FASN* and *SCD*, which are key genes in *de novo* fatty acid synthesis, suggesting that *FSCN1* promoted CRC by affecting lipid metabolism.

This regulatory action of *FSCN1* may be because CRC is closely related to obesity and lipid metabolism [39, 40]. Fatty acids are indispensable for the biosynthesis of most lipids, such as membrane lipids and lipid signaling molecules, in addition to acting as substrates for energy production [41]. *De novo* synthesis of palmitic acid from acetyl-CoA (acetyl-coenzyme A) is mainly catalyzed by fatty acid synthase (*FASN*). SFAs are converted into MUFAs by stearoyl-CoA desaturase 1 (*SCD*), and their chains are elongated by elongases [28]. A high-fat diet increases palmitic acid levels, which in turn increase *β*2AR expressions in a Sp-1 dependent manner. Subsequently, the cAMP/PKA axis is activated and hormone sensitive lipase (HSL) is phosphorylated at S552 to increase energy production, which promotes CRC growth [42]. In addition, there may be another *FSCN1*-lipid metabolism axis independent of LYAR, which would require future studies to confirm.

In summary, we demonstrated that *FSCN1* is a direct target of LYAR. A novel pathway for function of the transcription factor LYAR in CRC has also been revealed: LYAR promotes tumor migration and invasion by upregulating *FSCN1* expression, which in turn positively regulates fatty acid metabolism. These findings suggest that LYAR could be used as a prognostic and therapeutic candidate target for the prevention and treatment of CRC.

## Figures and Tables

**Table 1:** LYAR is highly expressed in human CRC tissues.

	Total	Positive (LYAR high expression)	Positive rate	*χ* ^2^	*p*
Adjacent	166	8	4.8%	81.806	<0.001
CRC	166	78	47.0%		

## Data Availability

The data generated or analyzed during the current study are available from the corresponding author on reasonable request.

## Supplementary Materials

Supplementary Materials Figure S1: Overall survival curve of those with colorectal cancer. Data were obtained from TCGA database and analyzed to generate Kaplan-Meier curves, which show a correlation between patients with tumors expressing low levels of LYAR and higher survival rates. Figure S2: LYAR expression in stable *LYAR* knockdown HCT15 cells. (a) RT-qPCR analysis of *LYAR* gene expression normalized to *GAPDH* gene expression in stable *LYAR* knockdown HCT15 cells. ^∗∗^*p* < 0.01 compared with the scrambled control. (b) Western blot assay showing LYAR protein expression in stable *LYAR* knockdown HCT15 cells. GAPDH served as the loading control. Figure S3: Detection of tumor cell phenotype in transient *LYAR* knockdown HCT15 cells. *LYAR* knockdown had no effect on cell proliferation (a), cell adhesion (b), cell cycle (c), apoptosis (d), or colony formation (e) of HCT15 cells. Parental refers to the control, nonsilencing refers to cells transfected with a nonspecific siRNA, and *LYAR*-siRNA1 and *LYAR*-siRNA2 refer to cells transfected with *LYAR*-specific siRNA1 and *LYAR*-specific siRNA2, respectively. Figure S4: Detection of migration and invasion in stable *LYAR* knockdown HCT15 cells. (a) Representative photos of haptotactic migration assay and matrigel chemoinvasion assay using stable *LYAR* knockdown HCT15 cells. Original magnification, 200x. (b) Results of migration and invasion assays. Data shown are the mean ± standard deviation (*n* = 3). ^∗∗^*p* < 0.01 compared to the scrambled control. Figure S5: Western blot assay showing FSCN1 expression after *LYAR* knockdown by siRNA. (a) LYAR and FSCN1 expression after *LYAR* knockdown in HCT8 cells. (b) LYAR and FSCN1 expression after *LYAR* knockdown in HCT116 cells. KD1 and KD2 refer to the stable *LYAR* knockdown lines *LYAR*-siRNA1 and *LYAR*-siRNA2, respectively. Figure S6: Expression of *LYAR* and downstream genes in colorectal cancer. The expression profiles of human colorectal cancer and normal colon tissue were obtained from TCGA and GTEx databases, respectively. The data show differential expression of *LYAR* (a), *FSCN1* (b), *FASN* (c), and *SCD* (d) in colorectal cancer tissues compared to adjacent normal tissues. ^∗∗∗^*p* < 0.001 and ^∗∗∗∗^*p* < 0.0001. Table S1: The relationship between LYAR protein expression and clinicopathological features in CRC patients. Table S2: ChIP primer sequences for the *FSCN1* promoter.
