# Supplementary material for: LYAR Promotes Colorectal Cancer Progression by Upregulating FSCN1 Expression and Fatty Acid Metabolism
Source: Oxid Med Cell Longev. 2021 Dec 6;2021:9979707. doi: 10.1155/2021/9979707 (PMC8769847; doi:10.1155/2021/9979707)
Supplement: Supplementary Materials — Figure S1: Overall survival curve of those with colorectal cancer. Data were obtained from TCGA database and analyzed to generate Kaplan-Meier curves, which show a correlation between patients with tumors expressing low levels of LYAR and higher survival rates. Figure S2: LYAR expression in stable LYAR knockdown HCT15 cells. (a) RT-qPCR analysis of LYAR gene expression normalized to GAPDH gene expression in stable LYAR knockdown HCT15 cells. ∗∗p < 0.01 compared with the scrambled control. (b) Western blot assay showing LYAR protein expression in stable LYAR knockdown HCT15 cells. GAPDH served as the loading control. Figure S3: Detection of tumor cell phenotype in transient LYAR knockdown HCT15 cells. LYAR knockdown had no effect on cell proliferation (a), cell adhesion (b), cell cycle (c), apoptosis (d), or colony formation (e) of HCT15 cells. Parental refers to the control, nonsilencing refers to cells transfected with a nonspecific siRNA, and LYAR-siRNA1 and LYAR-siRNA2 refer to cells transfected with LYAR-specific siRNA1 and LYAR-specific siRNA2, respectively. Figure S4: Detection of migration and invasion in stable LYAR knockdown HCT15 cells. (a) Representative photos of haptotactic migration assay and matrigel chemoinvasion assay using stable LYAR knockdown HCT15 cells. Original magnification, 200x. (b) Results of migration and invasion assays. Data shown are the mean ± standard deviation (n = 3). ∗∗p < 0.01 compared to the scrambled control. Figure S5: Western blot assay showing FSCN1 expression after LYAR knockdown by siRNA. (a) LYAR and FSCN1 expression after LYAR knockdown in HCT8 cells. (b) LYAR and FSCN1 expression after LYAR knockdown in HCT116 cells. KD1 and KD2 refer to the stable LYAR knockdown lines LYAR-siRNA1 and LYAR-siRNA2, respectively. Figure S6: Expression of LYAR and downstream genes in colorectal cancer. The expression profiles of human colorectal cancer and normal colon tissue were obtained from TCGA and GTEx databases, respectively. The da [file 9979707.f1.docx]

**Supplementary data list:**

**Figure S1**

**
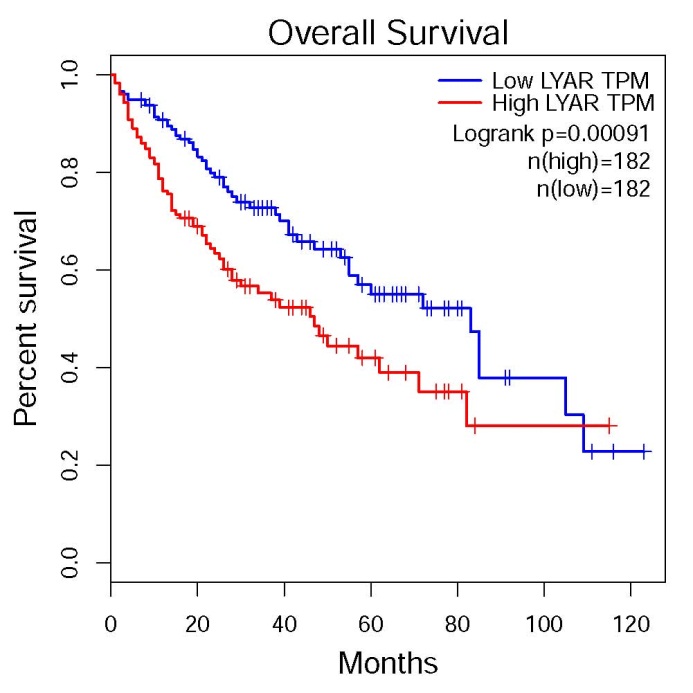
**

Figure S1: Overall survival curve of those with colorectal cancer. Data were obtained from the TCGA database and analyzed to generate Kaplan-Meier curves, which show a correlation between patients with tumors expressing low levels of LYAR and higher survival rates.

**Figure S2**

**
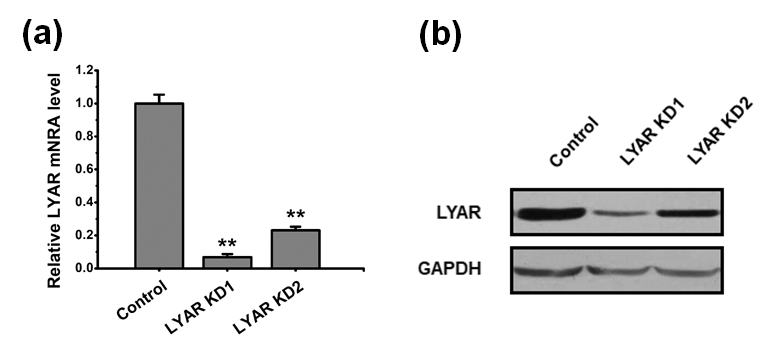
**

Figure S2: *LYAR* expression in stable *LYAR*-knockdown HCT15 cells. (a) RT-qPCR analysis of *LYAR* gene expression normalized to *GAPDH* gene expression in stable *LYAR*-knockdown HCT15 cells. ***p* < 0.01 compared with the scrambled control. (b) Western blot assay showing LYAR protein expression in stable *LYAR*-knockdown HCT15 cells. GAPDH served as the loading control.

**Figure S3**

**
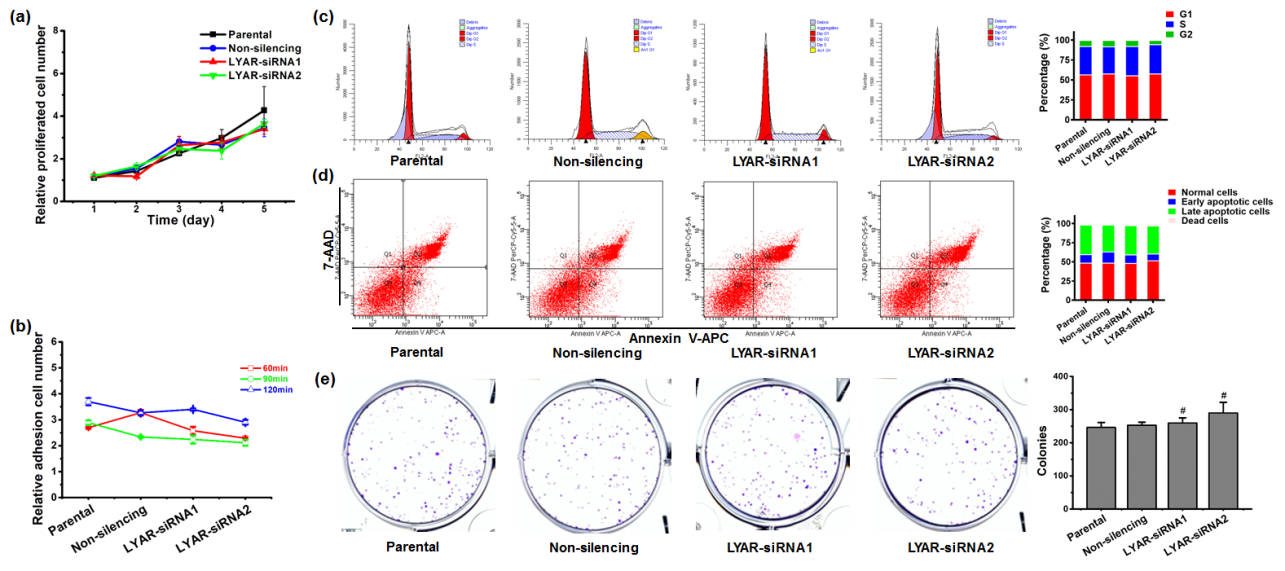
**

Figure S3: Detection of tumor cell phenotype in transient *LYAR*-knockdown HCT15 cells. *LYAR* knockdown had no effect on cell proliferation (a), cell adhesion (b), cell cycle (c), apoptosis (d), or colony formation (e) of HCT15 cells. Parental refers to the control, non-silencing refers to cells transfected with a nonspecific siRNA, and *LYAR*-siRNA1 and *LYAR*-siRNA2 refer to cells transfected with *LYAR*-specific siRNA1 and *LYAR*-specific siRNA2, respectively.

**Figure S4**


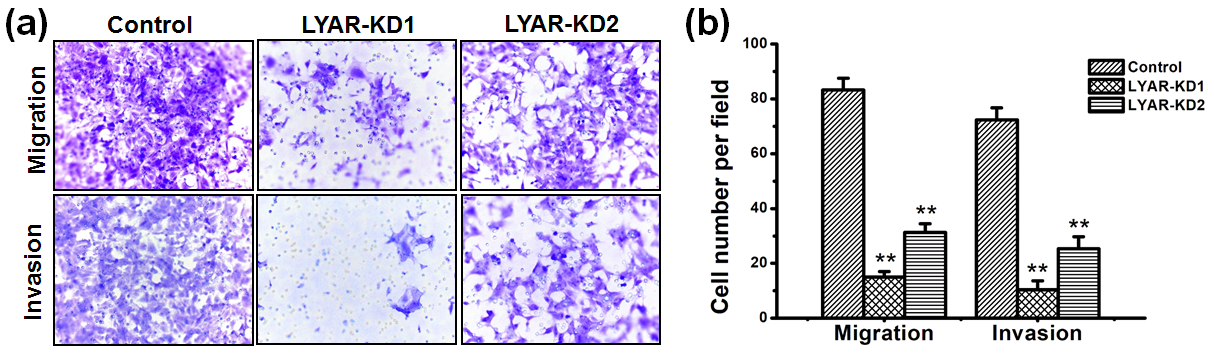


Figure S4: Detection of migration and invasion in stable *LYAR*-knockdown HCT15 cells. (a) Representative photos of haptotactic migration assay and matrigel chemoinvasion assay using stable *LYAR*-knockdown HCT15 cells. Original magnification, 200×. (b) Results of migration and invasion assays. Data shown are the mean ± standard deviation (n = 3). ***p* < 0.01 compared to the scrambled control.

**Figure S5**

**
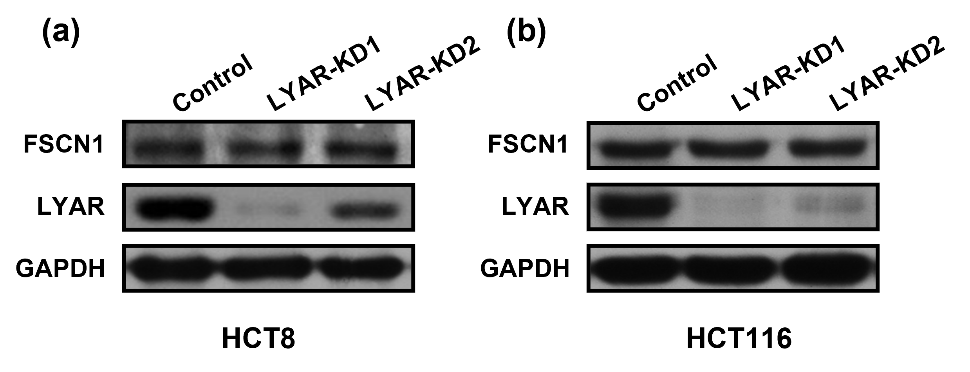
**

Figure S5: Western blot assay showing FSCN1 expression after *LYAR* knockdown by siRNA. (a) LYAR and FSCN1 expression after *LYAR* knockdown in HCT8 cells. (b) LYAR and FSCN1 expression after *LYAR* knockdown in HCT116 cells. KD1 and KD2 refer to the stable LYAR knockdown lines LYAR-siRNA1 and LYAR-siRNA2, respectively.

**Figure S6**


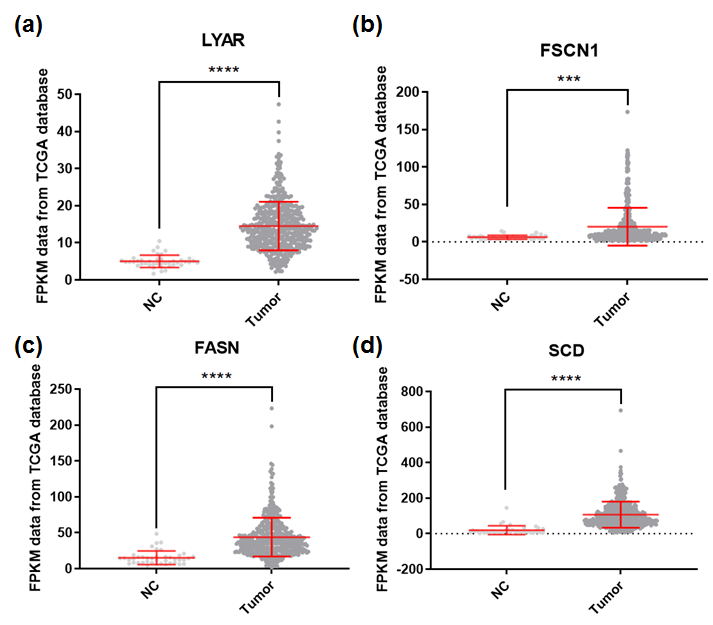


Figure S6: Expression of LYAR and downstream genes in colorectal cancer. The expression profiles of human colorectal cancer and normal colon tissue were obtained from the TCGA and GTEx databases, respectively. The data show differential expression of *LYAR* (**a**), *FSCN1* (**b**), *FASN* (**c**), and *SCD* (**d**) in colorectal cancer tissues compared to adjacent normal tissues. ****p* < 0.001, *****p* < 0.0001.

Table S1: The Relationship between LYAR protein expression and clinicopathological features in CRC patients.

| **Clinicopathological features** | **LYAR** | | ***P*** |
| --- | --- | --- | --- |
|  | **High** | **Low** |  |
|  | 78 | 88 |  |
| Gender |  |  |  |
| Male | 50 | 51 | 0.42 |
| Female | 28 | 37 |  |
| Age (years) |  |  |  |
| ≤60 | 39 | 39 | 0.46 |
| ＞60 | 39 | 49 |  |
| pT |  |  |  |
| T1-2 | 25 | 22 | 0.31 |
| T3-4 | 53 | 66 |  |
| pN |  |  |  |
| N0 | 47 | 54 | 0.88 |
| N1 | 31 | 34 |  |
| pM |  |  |  |
| M0 | 52 | 73 | 0.02 |
| M1 | 26 | 15 |  |
| AJCC7 stage |  |  |  |
| Ⅰ-Ⅱ | 33 | 48 | 0.12 |
| Ⅲ-Ⅳ | 45 | 40 |  |

**Notes**: pT, pathological stage; pN, lymph node metastases; pM, metastasis; AJCC7 stage, 7th Edition of the AJCC (American Joint Committee on Cancer) Cancer Staging Manual

Table S2: ChIP primer sequences for the *FSCN1* promoter.

| **Locus** | **Forward** | **Reverse** |
| --- | --- | --- |
| Primer 1: | CTCAGGGCAGAGCAAAGAAG | TGTCACCTTCTTGCCATCCT |
| Primer 2: | CATGTCCCTCCTGTGTGACC | GAGTGGGCAGGACAGGAATT |
| Primer 3: | CCGTGGTCTCTGAAGTCTCC | CCGCCTGAGGACATCACC |
| Primer 4: | CAGTTCGGCCTCATCAACTG | CACGATGAGGAAACGGCAG |
| Primer 5: | CGAGGAGTGGGGCTTTGC | GGAGGGGCACGAGGAAAT |
| Primer 6: | CCCTCACCCATTTCCTCGT | GAGGAGGTGGAGGAAGGC |
| Primer 7: | CAACTCGATCCGTCCCCTC | TGTCTGCAAGCAAGGAACAG |
